# Claudin-7 Modulates Cl^−^ and Na^+^ Homeostasis and WNK4 Expression in Renal Collecting Duct Cells

**DOI:** 10.3390/ijms20153798

**Published:** 2019-08-03

**Authors:** Junming Fan, Rodney Tatum, John Hoggard, Yan-Hua Chen

**Affiliations:** 1 Department of Anatomy and Cell Biology, Brody School of Medicine, East Carolina University, Greenville, NC 27834, USA; 2 Institute of Hypoxia Medicine, School of Basic Medical Sciences, Wenzhou Medical University, Wenzhou 325035, China; 3 East Carolina Diabetes and Obesity Institute, East Carolina University, Greenville, NC 27834, USA

**Keywords:** Claudin-7, tight junctions, permeability, WNK4, epithelial sodium channel (ENaC), collecting duct cells

## Abstract

Claudin-7 knockout (CLDN7^−/−^) mice display renal salt wasting and dehydration phenotypes. To address the role of CLDN7 in kidneys, we established collecting duct (CD) cell lines from CLDN7^+/+^ and CLDN7^−/−^ mouse kidneys. We found that deletion of CLDN7 increased the transepithelial resistance (TER) and decreased the paracellular permeability for Cl^−^ and Na^+^ in CLDN7^−/−^ CD cells. Inhibition of transcellular Cl^−^ and Na^+^ channels has no significant effect on TER or dilution potentials. Current-voltage curves were linear in both CLDN7^+/+^ and CLDN7^−/−^ CD cells, indicating that the ion flux was through the paracellular pathway. The impairment of Cl^−^ and Na^+^ permeability phenotype can be rescued by CLDN7 re-expression. We also found that WNK4 (its mutations lead to hypertension) expression, but not WNK1, was significantly increased in CLDN7^−/−^ CD cell lines as well as in primary CLDN7^−/−^ CD cells, suggesting that the expression of WNK4 was modulated by CLDN7. In addition, deletion of CLDN7 upregulated the expression level of the apical epithelial sodium channel (ENaC), indicating a potential cross-talk between paracellular and transcellular transport systems. This study demonstrates that CLDN7 plays an important role in salt balance in renal CD cells and modulating WNK4 and ENaC expression levels that are vital in controlling salt-sensitive hypertension.

## 1. Introduction

Maintaining electrolytes and body fluids across epithelial layers in kidneys within the physiological range is of vital importance for blood pressure regulation. Chloride (Cl^−^) and sodium (Na^+^), two predominant extracellular ionic components in kidneys, determine the extracellular electrolyte balance and regulate the blood pressure in the segment of the collecting duct (CD) [1]. It is well known that sodium reabsorption in the CD is an active process through the apical epithelial sodium channel (ENaC) and is driven by the basolateral Na^+^-K^+^-ATPase. On the other hand, chloride reabsorption is driven by the lumen-negative transepithelial potential and mainly occurs through tight junctions (TJs), the gatekeeper of the paracellular pathway in CD [1,2,3]. 

TJ is a multi-molecular complex and plays an essential role in regulating ions and small molecules passing through the apical to basal compartment of the epithelial cells [4,5]. Claudins (CLDN), a family of transmembrane proteins with at least 24 members in mouse and human, are the most important structural and functional components of the TJs and the principal regulators in defining the properties of paracellular ion permeability of the epithelial cells [6,7]. There are more than ten CLDN members expressed in kidneys, and they are closely associated with their corresponding segment-specific ion reabsorption characters [6,8]. Deficiency or aberrant expression of distinct CLDNs has been reported to be associated with disturbance of electrolytes, which can lead to high blood pressure or hypertension-related diseases. For example, mutations in CLDN16 and CLDN19 in humans resulted in kidney disorders exhibiting renal magnesium wasting and hypercalciuria [9,10]. Mice with CLDN16 knockdown exhibit defects in paracellular cation selectivity and develop severe renal wasting of magnesium and calcium [11]. A study by Muto et al. [12] demonstrated that CLDN2-deficient mice show a significant decrease in net transepithelial reabsorption of Na^+^ and Cl^−^ in proximal tubules, causing a loss of Na^+^ selectivity and therefore relative Cl^−^ selectivity in the proximal tubule paracellular pathway. Results from Krug et al. [13] reported that Madin-Darby Canine Kidney (MDCK) C7 cells with CLDN17 overexpression show an increase in paracellular anion permeability and switch from cation-selective to anion-selective. Knockdown of CLDN17 in LLC-PK1 cells support CLDN17 as an anion channel. In addition, mice with CLDN10 deletion in the thick ascending limb show the impairment in paracellular Na^+^ permeability and hypermagnesemia [14], and can rescue CLDN16-deficient mice from hypomagnesemia and hypercalciuria [15].

Pseudohypoaldosteronism type II (PHAII) is an autosomal-dominant hereditary hypertensive disease that is characterized by hyperkalemia and metabolic acidosis [16,17]. In 2001, Wilson et al. [18] found that the mutations in WNK4, a serine/threonine kinase with No K (lysine), were linked to the pathogenesis of PHAII. Since then, many studies have shown that several membrane channels and transporters are the molecular targets of WNK4 [16,19,20,21,22,23,24]. Since WNK4 is localized at the TJs of distal nephrons, TJ proteins could also serve as additional targets for WNK4. Indeed, two groups have reported that WNK4 regulates the paracellular Cl^−^ permeability in MDCK II cells [25,26], and the latest study documented by Chen et al. showed that mice with knockin Cl^−^-insensitive mutant WNK4 displayed hypertension, hyperkalemia, hyperactive NCC, and the authors concluded that WNK4 is a physiological intracellular Cl^−^ sensor [24]. Our previous study also showed that claudin-7 is the substrate of WNK4 and can be phosphorylated by WNK4 at serine^206^ in its COOH-terminus [27]. 

Although it is well characterized how transcellular channels and transports work in regulating Cl^−^ and Na^+^ transport in the CD of the kidney [12,14], the molecular targets of the paracellular pathway responsible for Cl^−^ and Na^+^ transport have not been fully elucidated. It has been reported that CLDN4 served as a Cl^−^ channel in mouse kidney CD cells that require the presence of CLDN8 [28]. We have previously reported that the first extracellular domain of CLDN7 affects paracellular Cl^−^ permeability [29]. In addition, overexpression of CLDN7 in LLC-PK1 cells decreased the paracellular Cl^−^ conductance and increased Na^+^ conductance [30]. 

To study the role of CLDN7 in vivo, we generated a CLDN7 knockout (CLDN7^−/−^) mouse model and discovered that CLDN7-deficient mice exhibited renal salt wasting, chronic dehydration, and severe intestinal defects, and that 90% of the pups died within 10 days after birth [31]. The kidney phenotypes suggest that CLDN7 plays an indispensable role in keeping salt homeostasis in distal nephrons. However, the functional role of CLDN7 in the distal nephron remains unclear. In this study, we isolated and purified CD cells from CLDN7^+/+^ and CLDN7^−/−^ mouse kidneys using Dolichos biflorus lectin-coated Dynabeads, and immortalized these cells into cell lines by Lenti-SV40 virus infection. These CD cells express AQP2, CLDN3, and CLDN4, but not CLDN2, a proximal tubule marker. We found that transepithelial resistance (TER) was significantly increased and paracellular Cl^−^ and Na^+^ permeability was decreased in CLDN7^−/−^ CD cells. These phenotypes can be rescued by the transfection of CLDN7 into CLDN7^−/−^ CD cells. In addition, we found in this study that WNK4 expression, but not WNK1, was significantly increased, and so was the ENaC level in CLDN7^−/−^ CD cells. These results suggest the potential influence of the paracellular pathway on transcellular pathway. Defects in the paracellular ion transport may affect transcellular transport systems, leading to the ionic imbalance in kidneys.

## 2. Results

### 2.1. Generation of CD Cell Lines Isolated from CLDN7^+/+^ and CLDN7^−/−^ Mouse Kidneys

**Figure 1:**
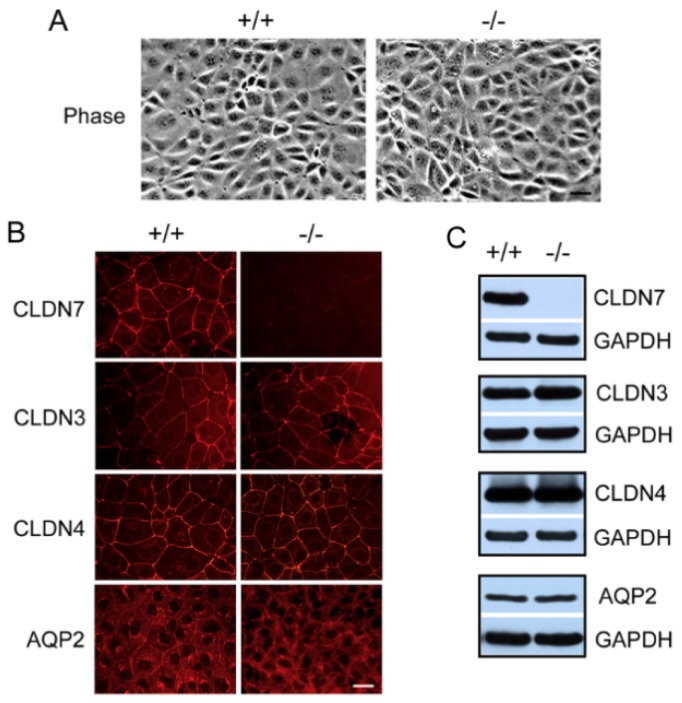
Characterization of immortalized mouse collecting duct (CD) cell lines. (**A**) Phase images of CLDN7^+/+^ (+/+) and CLDN7^−/−^ (−/−) CD cells. CD cells from 4-day old mouse CLDN7^+/+^ and CLDN7^−/−^ kidneys were isolated and purified by Dolichos biflorus lectin-coated Dynabeads. These purified CD cells were immortalized into cell lines using SV40 virus. The stable CD cells were grown on coverslips for 5–7 days before fixation for fluorescent light microcopy. Bar: 30 µm. (**B**) CLDN7^+/+^ and CLDN7^−/−^ CD cells were immunostained with antibodies against CLDN7, CLDN3, CLDN4, and AQP2 and detected by Cy3-conjugated anti-rabbit secondary antibody. CLDN7^+/+^ CD cells showed a strong anti-CLDN7 immunostaining and this signal was completely absent in CLDN7^−/−^ CD cells. Both CLDN3 and CLDN4 have similar staining patterns in CLDN7^+/+^ and CLDN7^−/−^ CD cells. AQP2 is a marker protein for CD principle cells and its membrane staining was much weaker in CLDN7^−/−^ than in CLDN7^+/+^ CD cells. Bar: 20 µm. (**C**) Western blot analysis confirmed the absence of CLDN7 protein in CLDN7^−/−^ CD cells. CLDN3, CLDN4, and AQP2 expression levels were similar between CLDN7^+/+^ and CLDN7^−/−^ CD cells. GAPDH signal was used as a loading control.

To study the role of claudin-7 in CDs, we generated CD cell lines isolated and purified from CLDN7^+/+^ and CLDN7^−/−^ mouse kidneys using Dolichos biflorus lectin-coated Dynabeads, and then immortalized these cells into multiple cell lines by Lenti-SV40 virus infection. The immortalized CLDN7^+/+^ and CLDN7^−/−^ CD cells have an epithelial morphology as shown in Figure 1A. CLDN7^+/+^ CD cells have a strong CLDN7 immunostaining signal localized at cell–cell contact area, while CLDN7 signal was absent in CLDN7^−/−^ CD cells as expected (Figure 1B, top panel). CLDN3, CLDN4, and AQP2 were known to be expressed in CD cells, and their presences were confirmed by both immunofluorescence microscopy and western blot analysis (Figure 1B,C). It was observed that AQP2 signal was quite weak at the cell membrane in CLDN7^−/−^ CD cells (Figure 1B); however, the protein expression level was similar between CLDN7^+/+^ and CLDN7^−/−^ CD cells (Figure 1C). CLDN8 signal was undetectable in both CLDN7^+/+^ and CLDN7^−/−^ CD cells (data unpublished). 

### 2.2. Decreased Paracellular Cl^−^ and Na^+^ Permeability in CLDN7^−/−^ CD Cells

**Figure 2:**
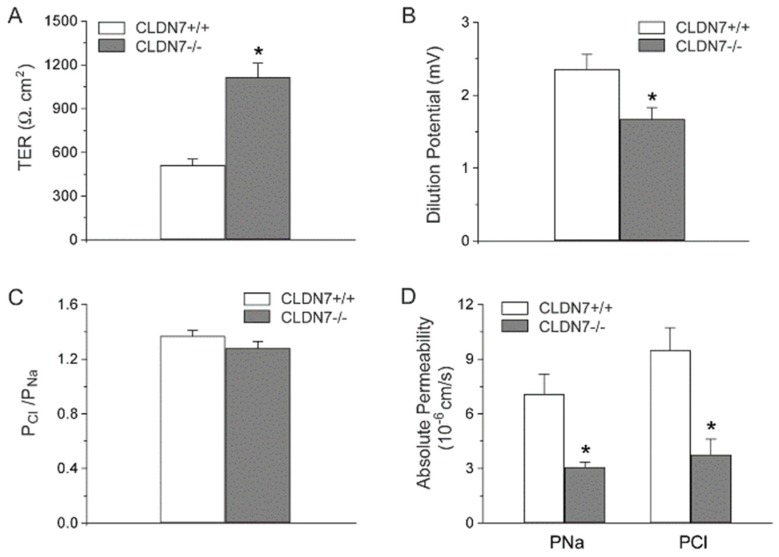
Deletion of CLDN7 increased transepithelial resistance (TER) and decreased paracellular Cl^−^ and Na^+^ permeability on CD cell monolayers. (**A**) TER was measured on monolayers cultured for 7 days. (**B**) CD cells were grown on collagen-coated Snapwell filters for 7 days to reach the full confluence. The filter rings containing cell monolayers were mounted into EasyMount chambers. Both apical and basal chambers were filled with buffer containing 140 mM NaCl. Subsequently, buffer in the basal chamber was replaced by 70 mM NaCl, and dilution potentials were measured. (**C**) The ratio of the absolute permeability of Cl^-^ to Na^+^ (P_Cl_/P_Na_) was calculated using the Goldman–Hodgkin–Katz equation. The ratio of P_Cl_/P_Na_ was >1 in CLDN7^+/+^, indicating that these CD cells were more permeable to Cl^−^ than Na^+^. (**D**) The absolute permeability for P_Cl_ and P_Na_ was calculated according to the method of simplified Kimizuka and Koketsu equations. * *p* < 0.05. *n* = 3.

To examine CLDN7-based CD cell electrophysiology, we first measured the TER of CLDN7^+/+^ and CLDN7^−/−^ CD cells. The TER of CLDN7^+/+^ CD cells has an average value of 510 ± 43 Ω.cm^2^. Deletion of CLDN7 dramatically increased TER to 1115 ± 96 Ω.cm^2^ (Figure 2A), suggesting an increase in barrier function induced by CLDN7 absence in CD cells. 

To further examine the ion permeability in our established CD cell lines, we performed dilution potential experiments. Our data showed that dilution potentials measured from CLDN7^−/−^ CD cells were significantly reduced compared to those of CLDN7^+/+^ CD cells (Figure 2B). However, the ratio of absolute permeability of Cl^−^ (P_Cl_) to Na^+^ (P_Na_) was slightly decreased for CLDN7^−/−^ CD cells, but without statistical significance (Figure 2C). Deletion of CLDN7 in CD cells depressed the permeation of Cl^−^ and Na^+^ as indicated by their reduced absolute permeability values of Cl^−^ (P_Cl_) and Na^+^ (P_Na_) (Figure 2D). Inhibition of epithelial Na^+^ and Cl^−^ channels had no significant effect on TER or dilution potentials either in CLDN7^+/+^ or CLDN7^−/−^ CD cells, indicating that the impairment of Cl^−^ and Na^+^ permeability in CLDN7^−/−^ CD cells is through the paracellular pathway (data unpublished). Moreover, current–voltage curves were linear in both CLDN7^+/+^ and CLDN7^−/−^ CD cells, consistent with the conductance being attributable to the paracellular pathway for ion flux (data unpublished). Our results indicate that CLDN7 plays a vital role in NaCl reabsorption in mouse CD cells. Deletion of CLDN7 decreases paracellular permeability to Cl^−^ and Na^+^, suggesting CLDN7 may serve as a non-selective paracellular channel in CD cells.

### 2.3. Increased Expression Levels of WNK4 and ENaC in CLDN7^−/−^ CD Cells

**Figure 3:**
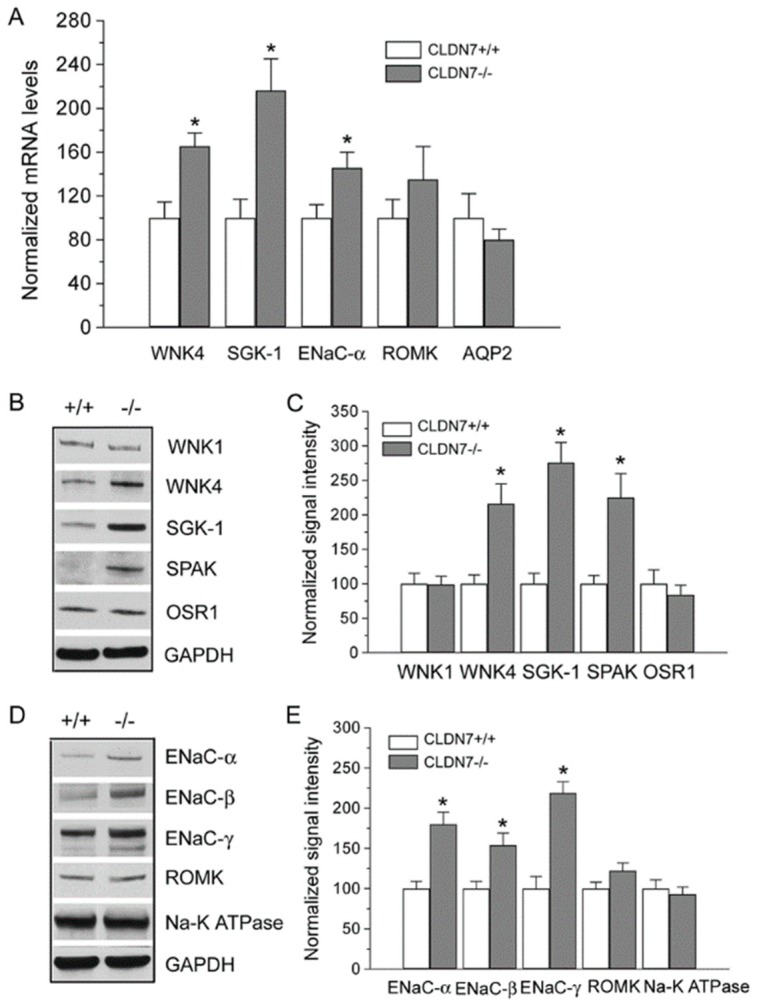
Deletion of CLDN7 had a significant effect on gene and protein expression levels of WNK4, SGK-1, and ENaC. (**A**) Real-time RT-PCR analysis of WNK4, SGK-1, ENaC-α, ROMK, and AQP2 mRNA levels in CLDN7^+/+^ and CLDN7^−/−^ CD cells. Each measurement was normalized to its β-actin level. * *p* < 0.05. *n* = 3. (**B**) Western blotting analysis of several protein kinase levels in CD cells. CLDN7^+/+^ and CLDN7^−/−^ CD cells were lysed in RIPA (radio-immunoprecipitation assay) buffer and a total of 30 μg protein for each lane was loaded onto the SDS NuPAGE gel. Membranes were blotted against WNK1, WNK4, SGK-1, SPARK, and OSR1. GAPDH (glyceraldehyde 3-phosphate dehydrogenase) staining was used as a loading control. (**C**) Densitometry analysis of protein expression levels shown on (**B**). Each band intensity for CLDN7^+/+^ CD cells was normalized and set as a reference. * *p* < 0.05. *n* = 3. (**D**) Western blotting analysis of several ion channel levels in CD cells. Equal amounts of CLDN7^+/+^ and CLDN7^−/−^ CD cell lysates were loaded onto the SDS NuPAGE gel and the membranes were probed against ENaC-α, -β, -γ, ROMK, and Na^+^-K^+^-ATPase. (**E**) Densitometry analysis of protein expression levels shown on (**D**). Each band intensity for CLDN7^+/+^ CD cells was normalized and set as a reference. * *p* < 0.05. *n* = 3.

We reported previously that CLDN7 was colocalized with WNK4 in kidneys and that they formed a protein complex when co-expressed in kidney epithelial cells [27]. To investigate whether CLDN7 deletion affects the expression of WNK4 and other kinases and ion channels, we performed real-time RT-PCR experiments. We found that deletion of CLDN7 significantly increased WNK4, SGK-1, and ENaC-α mRNA levels, while there were no significant changes in ROMK and AQP2 mRNA levels (Figure 3A). 

**Figure 4:**
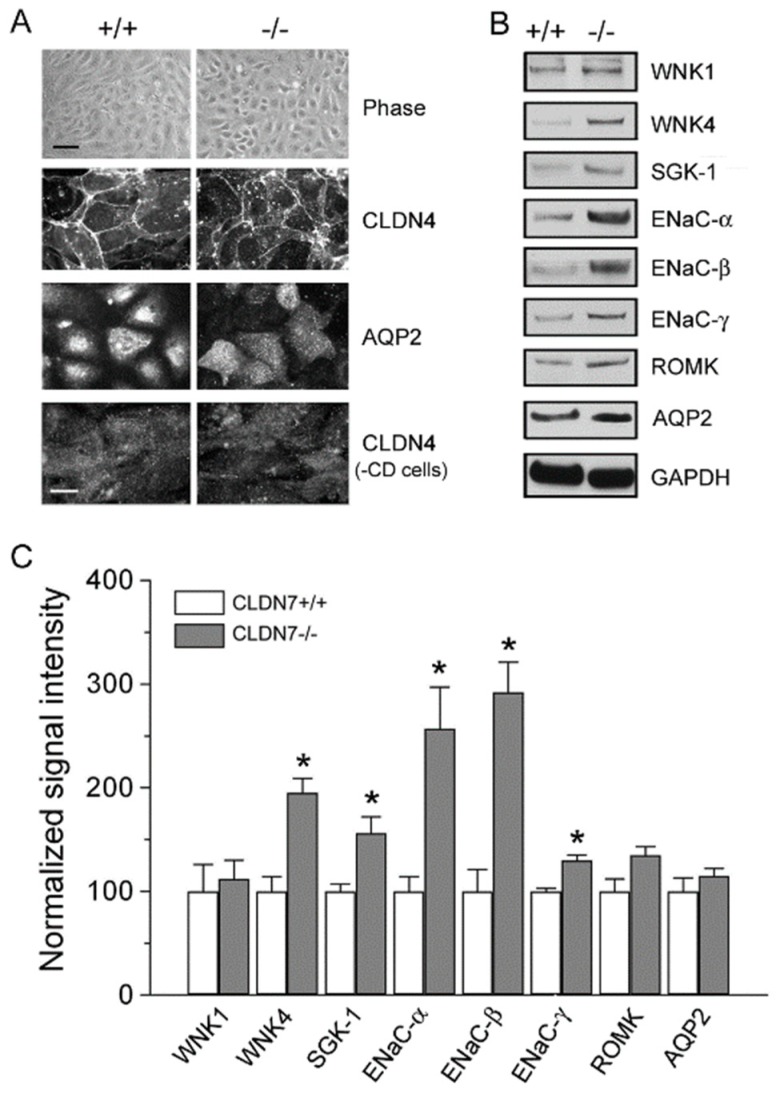
Deletion of CLDN7 had a significant effect on protein expression levels of representative kinases and ion channel on primary CD cells. (**A**) The establishment of primary cultures of CD cells isolated from kidneys of CLDN7^+/+^ and CLDN7^−/−^ pups. The top panel shows the phase images of primary CD cells isolated from kidneys of 5-day old CLDN7^+/+^ and CLDN7^−/−^ pups after cultured in 12-well plates for a week to form a complete monolayer. Bar: 40 µm. The cultured primary CLDN7^+/+^ and CLDN7^−/−^ CD cells were immunostained with anti-CLDN4 and anti-AQP2 antibodies. The last panel shows the remaining cells immunostained with anti-CLDN4 antibody after removal of CD cells. Bar: 15 µm. (**B**) The primary CD cells were lysed in RIPA buffer and a total of 30 μg protein for each lane was loaded onto the SDS NuPAGE gel. Membranes were blotted against WNK1, WNK4, SGK-1, ENaC-α, -β, and -γ, ROMK, and AQP2. GAPDH was used as a loading control. (**C**) Densitometry analysis of protein expression levels shown on (**B**). Each band intensity for CLDN7^+/+^ CD cells was normalized and set as a reference. * *p* < 0.05. *n* = 4.

Immunoblotting analysis also showed that the protein expression levels of WNK4, SGK-1, and SPAK were all clearly increased, but WNK1 and OSR1 levels were unchanged in CLDN7^−/−^ CD cells compared to those in CLDN7^+/+^ CD cells (Figure 3B,C). Interestingly, we found that the expression levels of ENaC-α, -β and –γ were all elevated with no changes in ROMK and Na-K-ATPase in CLDN7^−/−^ CD cells (Figure 3D,E). We have confirmed these results in the primary CLDN7^+/+^ and CLDN7^−/−^ CD cells as shown in Figure 4. The phase images of primary CD cells isolated from CLDN7^+/+^ and CLDN7^−/−^ kidneys were shown in Figure 4A (top panel). Anti-CLDN4 and anti-AQP2 antibodies were used to stain CD cells (Figure 4A). After CD cells were removed, the remaining cells were immunostained with CLDN4 and found to be CLDN4-negative (Figure 4A, bottom panel). Consistent with the immortalized CLDN7^−/−^ CD cells, CLDN7 deletion clearly increased WNK4, SGK-1, and ENaC subunit’s expression levels with no significant effects on WNK1, ROMK, or AQP2 expression levels in primary CLDN7^−/−^ CD cells (Figure 4B,C). 

### 2.4. Rescued Function of Ion Permeability in Immortalized CLDN7^+/+^ CD Cells with CLDN7 Knockdown

**Figure 5:**
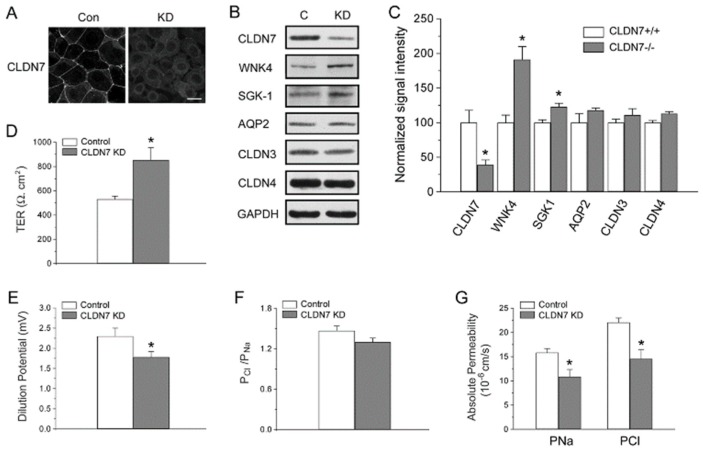
Knockdown of CLDN7 in CLDN7^+/+^ CD cells decreased paracellular Cl^−^ and Na^+^ permeability. (**A**) CLDN7^+/+^ CD cells were transfected with either control (Con) or CLDN7 shRNA (KD) vector. The indirect immunofluorecent method shows the reduced immunostaining signal of CLDN7 in KD cells compared to that of control cells. (**B**) The control and knockdown (KD) cell lysates were subject to western blot analysis. Membranes were probed against CLDN7, WNK4, SGK-1, AQP2, CLDN3, and CLDN4. GAPDH was used as a loading control. (**C**) Densitometry analysis of protein expression levels shown on (**B**). Each band intensity for CLDN7^+/+^ CD cells was normalized and set as a reference. * *p* < 0.05. *n* = 3. (**D**) The control and KD CD cells were cultured in Transwell plates coated with collagen. TER was measured on monolayers cultured for 7 days. (**E**) The control and KD CD cells were grown on collagen-coated Snapwell filters for 7 days. The dilution potentials were measured as described in Figure 2B. (**F**) The ratio of the absolute permeability of Cl^-^ to Na^+^ (P_Cl_/P_Na_) was calculated using the Goldman–Hodgkin–Katz equation. (**G**) The calculated absolute permeability for P_Cl_ and P_Na_ was significantly reduced in KD cells compared to that of control cells. * *p* < 0.05. *n* = 3.

As we observed an increase in barrier function and a decrease in Cl^−^ and Na^+^ permeability in CLDN7^−/−^ CD cells, we tried to stably transfect CLDN7 back (‘rescue’) into CLDN7^−/−^ CD cells to study whether CLDN7 could revert the phenotype. However, we were unable to obtain the stable cell lines after many attempts. Therefore, herein we designed specific shRNAs to knock down the expression of CLDN7 in CLDN7^+/+^ CD (KD) cells and then transfected CLDN7 back into these KD cells. Immunofluorescent staining and western blot analysis confirmed the knockdown of the expression of CLDN7 in CLDN7^+/+^ CD cells by CLDN7 shRNA (Figure 5A–C). Similarly as in CLDN7^−/−^ CD cells, CLDN7 KD induced an increase in WNK4 and SGK-1 expression while AQP2, CLDN3, and CLDN4 expressions were unchanged (Figure 5B,C). In addition, we found that CLDN7 KD also significantly increased the TER value by 61.2% compared with the scrambled controls (Figure 5D). Moreover, CLDN7 KD decreased dilution potential (DP) (Figure 5E) and Cl^−^ and Na^+^ permeability (Figure 5G) as we found in CLDN7^−/−^ CD cells without a significant change in PCl/PNa (Figure 5F). 

**Figure 6:**
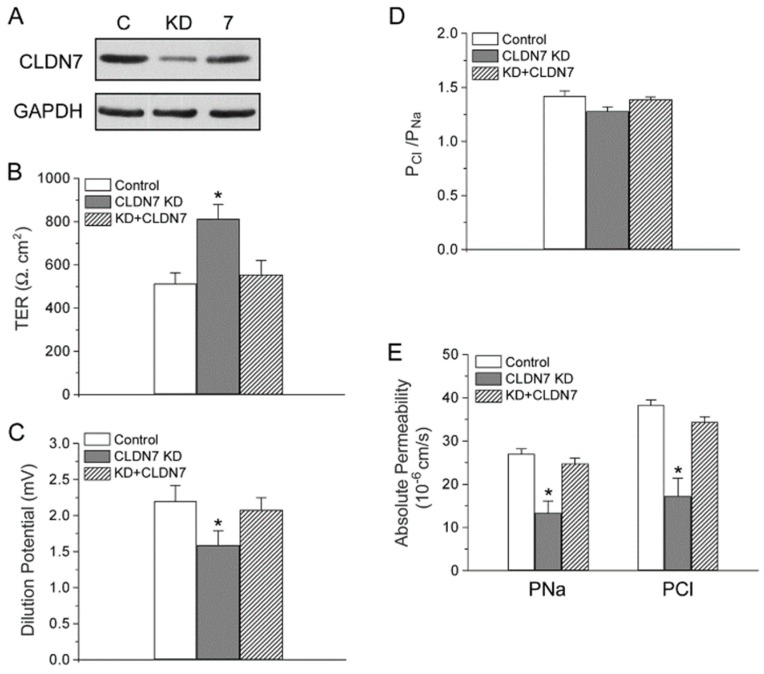
Re-expression (‘rescue’) of CLDN7 in CLDN7^+/+^ CD cells with CLDN7 knockdown restored paracellular Cl^−^ and Na^+^ permeability. (**A**) CD cells from control (C), CLDN7 knockdown (KD), KD with CLDN7 cDNA transfection (7) were lysed in RIPA buffer and subjected to western blotting. The membrane was blotted with anti-CLDN7 antibody. (**B**) TER values were measured on cell monolayers cultured for 7 days on collagen-coated Transwell plates. (**C**) Dilution potentials were measured as described in Figure 2B. (**D**) The ratio of the absolute permeability of Cl^−^ to Na^+^ (P_Cl_/P_Na_), and (**E**) the absolute permeability for P_Cl_ and P_Na_ were calculated as described in Figure 2C,D, respectively. * *p* < 0.05 compared to control and KD+CLDN7. *n* = 3.

Transfection of CLDN7 back to the CLDN7^+/+^ KD cells (KD+CLDN7) increased the protein expression of CLDN7 to 88.2% of the control cell value (Figure 6A). The TER and DP were also back to the values similar to those in control cells (Figure 6B,C). Although there was no significant difference in PCl/PNa among the control, KD, and KD+CLDN7 CD cells (Figure 6D), Cl^−^ and Na^+^ permeability was recovered to 91.1% and 90.4% in CLDN7 rescued cells, respectively (Figure 6E). 

## 3. Discussion

In this study, we have shown that mouse renal CD cells with CLDN7 deletion exhibited significant decrease in paracellular Cl^−^ and Na^+^ permeability. At the same time, the TER in CLDN7^−/−^ CD cells was greatly increased while the dilution potential was decreased. The paracellular ion permeability in CLDN7^+/+^ CD cells with CLDN7 knockdown resembled that of CLDN7^−/−^ CD cells. Re-expression (‘rescue’) CLDN7 in CLDN7^+/+^ KD cells restored the role of CLDN7 in paracellular Cl^−^ and Na^+^ permeability. In addition, our study demonstrates that deletion of CLDN7 upregulates WNK4 expression at both mRNA and protein levels in our immortalized CD cells as well as in the primary CD cells, indicating that CLDN7 may play an important role in regulating WNK4 expression in kidneys. Interestingly, ENaC expression was also upregulated in immortalized and primary CLDN7^−/−^ CD cells compared to that of CLDN7^+/+^ CD cells, suggesting a potential influence of paracellular pathway on transcellular pathway.

It is known that CLDN7 is highly expressed in the distal nephron of the kidney [32]. We reported previously that CLDN7-deficient mice exhibited renal salt wasting and chronic dehydration [31]. To investigate the function of CLDN7 in distal nephron, we used the novel approach to isolate, purify, and immortalize the CD cells from CLDN7^+/+^ and CLDN7^−/−^ mouse kidneys. This approach allows us to study the role of CLDN7 in renal epithelial cells in a controlled environment without the stimulation of hormones and other circulating factors. We found that the TER value was around 500 Ω.cm^2^ for CLDN7^+/+^ CD cells, which was consistent with the literature on isolated rabbit CDs [33]. However, deletion of CLDN7 increased TER value to more than 1000 Ω.cm^2^. The increase in TER is due to the decrease in paracellular Cl^−^ and Na^+^ permeability. We reported previously that overexpression of CLDN7 in LLC-PK1 cells decreases the paracellular Cl^−^ conductance and increases paracellular Na^+^ conductance [30]. It is possible that the effect of overexpression or knockdown of the same gene in different cell lines may have different functional consquences as we have observed in human lung cancer cells [34] and our unpublished data. It is known that the paracellular pathway in the CD system is mainly Cl^−^ selective [1,35]. However, Cl^−^ reabsorption must match that of Na^+^ in order to maintain the homeostasis of luminal fluids and electrolytes. Our current study suggests that CLDN7 may form a non-selective paracellular channel in renal CD cells and play a critical role in Cl^−^ and Na^+^ homeostasis in distal nephrons. 

WNK4 is localized at TJs of distal nephrons and has been shown to selectively increase paracellular Cl^−^ permeability and phosphorylate claudins in MDCK cells [18,25,26], and many studies have revealed that mutations of WNK4 are involved in the pathogenesis of PHAII [18,24,36,37]. We previously found that CLDN7 was a substrate of WNK4, and that phosphorylation of CLDN7 by WNK4 promoted the paracellular Cl^−^ permeability in kidney epithelial cells [27]. Interestingly, we found in this study that deletion of CLDN7 significantly increased WNK4 expression in CD cell lines as well as in primary CD cells (Figure 3B and Figure 4B), suggesting a previously unrecognized involvement of CLDN7 in the regulation of WNK4. In addition, ENaC expression was also increased in both CLDN7^−/−^ immortalized and primary CD cells, which could be mediated through the up-regulation of SGK-1 since it has been reported that SGK-1 stimulates the membrane expression and the activity of ENaC [38,39,40]. It will be interesting to see in future studies whether ENaC channel activity is altered in CLDN7^−/−^ CD cells. 

It has been reported that CLDN4 forms a paracellular Cl^-^ channel in the kidney and requires CLDN8 for TJ localization [28]. However, in our CLDN7 CD cell lines, CLDN4 was well localized to the cell junction area in both CLDN7^+/+^ and CLDN7^−/−^ CD cells, though the CLDN8 signal was undetectable. It is possible that different renal epithelial cells may behave differently depending on the TJ components and claudin compositions. 

## 4. Materials and methods

### 4.1. Antibodies and Reagents

Rabbit polyclonal anti-CLDN3 and CLDN4 antibodies were purchased from Invitrogen (Thermo Fisher Scientific, Waltham, MA, USA). Rabbit polyclonal anti-CLDN7 antibody was obtained from Immuno-Biological Laboratories (Gunma, Japan). AQP2 polyclonal antibody was purchased from CALBIOCHEM (Sigma-Aldrich, St. Louis, MO, USA). Rabbit anti-WNK4 antibody was described previously [41]. The WNK1 antibody was obtained from Novus Biologicals (Centennial, CO, USA). Anti-SGK-1 and Anti-SPAK antibodies were from Cell Signaling Technology (Danvers, MA, USA). OSR1 antibody was purchased from Abcam (Cambridge, MA, USA). All chemicals and reagents were purchased from Sigma and/or Fisher Scientific unless noted otherwise. Transwell and Snapwell plates were from Corning Costar (Corning, NY, USA).

### 4.2. Isolation and Immortalization of CD Cells from CLDN7^+/+^ and CLDN7^−/−^ Mouse Kidneys

The kidneys were removed quickly from 4–5-day-old CLDN7^+/+^ and CLDN7^−/−^ mice generated in this laboratory [31], minced into 1 mm^3^ pieces, and digested in Hanks’ Balanced Salt Solution (HBSS, Invitrogen, ThermoFisher Scientific, Waltham, MA USA) containing 0.2% collagenase A and 0.2% hyaluronidase (Sigma, St. Louis, MO, USA). After 30 min incubation at 37 °C, DNase I (100 U/mL) was added to the cell suspension to prevent cell clumping. Then the cell suspension went through a cell strainer and the collected cells were incubated with Dolichos biflorus lectin-coated Dynabeads (Invitrogen, ThermoFisher Scientific) in a tube at 8 °C for 20 min. The tube was placed in a magnet for 2 min and the supernatant was discarded. The beads-bound cells were washed with PBS, separated from the supernatant by the magnet, and re-suspended in an established renal CD medium [42]. To release the cells from the beads, DNase was added to the tube containing beads-bound cells. The released cells and beads were separated by the magnet, and the supernatant with released cells were cultured in DMEM/Ham’s F12 medium containing 5% fetal bovine serum, 2.5 μg/mL transferrin, 1 μM thyronine T_3_, 30 μM sodium selenate, 2 mM L-glutamine, 15 mM HEPES, 100 U/mL penicillin, and 100 μg/mL streptomycinneomycin in a humidified 5% CO_2_-air atmosphere at 37 °C. The purified CD cells were immortalized into cell lines using Lenti-SV40 virus infection according to the manufacturer’s instructions (Applied Biological Materials Inc. Richmond, Canada). At least three CD cell lines were established by the above methods and used in the current study. All animal experiments were approved by the East Carolina University Animal Care and Use Committee (AUP#A172b, date of approval 16 December 2011).

### 4.3. Electrophysiological Measurements

*Transepithelial resistant (TER) measurements:* CLDN7^+/+^ and CLDN7^−/−^ CD cells were plated onto collagen-coated Transwell inserts at the density of 1.5 × 10^5^ cells/cm^2^ and cultured for 7–10 days. After the monolayer reached confluence, the resistance across each filter was measured by a Millicell-ERS Volt-Ohm meter (Millipore, Bedford, MA, USA). All TER values were calculated by subtracting the resistance measured in the blank insert from the resistance measured in the insert with the monolayer and then multiplied by the surface area of the membrane.

*Dilution potential measurements:* CLDN7^+/+^ and CLDN7^−/−^ CD cells were grown on Snapwell membranes coated with collagens. The apical and basal chambers were filled with P1 buffer containing (in mM): 140 NaCl, 2 CaCl_2_, 1 MgCl_2_, 10 HEPES, and 10 glucose (pH 7.3). To measure the dilution potential (DP), the basal chamber was switched from P1 to P2 buffer containing (in mM): 70 NaCl, 140 mannitol, 2 CaCl_2_, 1 MgCl_2_, 10 HEPES, and 10 glucose (pH 7.3). During the experiments, the buffer was maintained at 37 °C and bubbled constantly with 95% air and 5% CO_2_. Dilution potential measurements and calculations were conducted as described in Alexandre et al. [30] and Yu et al. [43]. Briefly, the ion permeability ratio of the monolayer to Cl^−^ over the permeability to Na^+^ (β = P_Cl_/P_Na_) was calculated from the dilution potential using the Goldman–Hodgkin–Katz equation. The absolute permeability values of Na^+^ (P_Na_) and Cl^−^ (P_Cl_) were calculated according to the following equations, P_Na_ = G · (RT/F^2^)/(α(1 + β) and P_Cl_ = P_Na_ · β. Here, the conductance per unit surface area (G) of the membrane can be measured by Ohm’s law, α is the NaCl activity, and β is the ratio of the permeability of Cl^−^ to that of Na^+^ as determined by the Goldman–Hodgkin–Katz equation [43]. Amiloride (100 µM, Sigma, St Louis, MO, USA), niflumic acid (NFA, 100 µM), and 4,4′-diisothiocyanatostilbene-2,2′-disulfonic acid (DIDS, 100 µM) were added to the solution to block epithelial sodium and chloride channels in CD cells.

### 4.4. RNA Extraction and Quantitative Real-Time PCR

The total RNA of CLDN7^+/+^ and CLDN7^−/−^ CD cells were isolated using a Qiagen RNeasy kit (Qiagen, Valencia, CA, USA), and the first-strand cDNA was synthesized with a Qiagen First Strand Kit according to the manufacturer’s instructions. Quantitative real-time RT-PCR (qRT-PCR) was performed as previously described [44]. For each target gene, the relative gene expression was performed in triplicates and the cycle threshold (Ct) values were normalized to the internal control β-actin gene expression level and analyzed by 2^−ΔΔ*C*t^ method [45].

### 4.5. Statistical Analysis

Statistical analysis was performed using Origin50 and VassarStats programs. The differences between two groups were analyzed using the unpaired two-tailed Student’s t-test. One-way ANOVA was performed if comparisons involved more than two groups. Data are expressed as mean ± S.E.M. and *n* indicates the number of independent experiments. A *p*-value of <0.05 was considered significant.

## 5. Conclusion

In conclusion, our present study highlights a critical role of CLDN7 in Cl^−^ and Na^+^ homeostasis in CD cells of kidneys and the involvement of CLDN7 in WNK4 regulation. In addition, the increased expression of ENaC in stable and primary CLDN7^−/−^ CD cells suggests the influence of an altered paracellular pathway on the transcellular pathway. Future studies should involve the functional analysis of ENaC channel activity in the presence and absence of CLDN7. Therefore, our mouse CD cell lines provide a unique model for investigating the crosstalk between paracellular and transcellular pathways and how this interaction affects the ionic balance of the kidney and blood pressure in the body.
